# Low Spontaneous Mutation Rate in Complex Multicellular Eukaryotes with a Haploid–Diploid Life Cycle

**DOI:** 10.1093/molbev/msad105

**Published:** 2023-05-04

**Authors:** Marc Krasovec, Masakazu Hoshino, Min Zheng, Agnieszka P Lipinska, Susana M Coelho

**Affiliations:** Sorbonne Université, CNRS, UMR 7232 Biologie Intégrative des Organismes Marins (BIOM), Observatoire Océanologique, Banyuls-sur-Mer, France; Department of Algal Development and Evolution, Max Planck Institute for Biology Tübingen, Tübingen, Germany; Department of Algal Development and Evolution, Max Planck Institute for Biology Tübingen, Tübingen, Germany; Department of Algal Development and Evolution, Max Planck Institute for Biology Tübingen, Tübingen, Germany; Department of Algal Development and Evolution, Max Planck Institute for Biology Tübingen, Tübingen, Germany

**Keywords:** mutation rate, spontaneous mutation, brown algae, chromosome duplication, life cycle

## Abstract

The spontaneous mutation rate *µ* is a crucial parameter to understand evolution and biodiversity. Mutation rates are highly variable across species, suggesting that *µ* is susceptible to selection and drift and that species life cycle and life history may impact its evolution. In particular, asexual reproduction and haploid selection are expected to affect the mutation rate, but very little empirical data are available to test this expectation. Here, we sequence 30 genomes of a parent–offspring pedigree in the model brown alga *Ectocarpus* sp.7, and 137 genomes of an interspecific cross of the closely related brown alga *Scytosiphon* to have access to the spontaneous mutation rate of representative organisms of a complex multicellular eukaryotic lineage outside animals and plants, and to evaluate the potential impact of life cycle on the mutation rate. Brown algae alternate between a haploid and a diploid stage, both multicellular and free living, and utilize both sexual and asexual reproduction. They are, therefore, excellent models to empirically test expectations of the effect of asexual reproduction and haploid selection on mutation rate evolution. We estimate that *Ectocarpus* has a base substitution rate of *µ*_bs_ = 4.07 × 10^−10^ per site per generation, whereas the *Scytosiphon* interspecific cross had *µ*_bs_ = 1.22 × 10^−9^. Overall, our estimations suggest that these brown algae, despite being multicellular complex eukaryotes, have unusually low mutation rates. In *Ectocarpus*, effective population size (*N*_e_) could not entirely explain the low *µ*_bs_. We propose that the haploid–diploid life cycle, combined with extensive asexual reproduction, may be additional key drivers of the mutation rate in these organisms.

## Introduction

The spontaneous mutation rate (*µ*) dictates the amount of new genetic diversity generated in a population or genome each generation, and reflects the fidelity of genome transmission. The importance of mutations has been recognized for more than a century, and great efforts have been made to understand the mechanisms underlying mutations and mutation rate evolution (Morgan 1910; Muller 1928; Demerec 1937; Haldane 1937). In the 1960s, Mukai statistically estimated the rate of deleterious mutations in *Drosophila* (Mukai 1964; Keightley and Eyre-Walker 1999) using the decrease of fitness during mutation accumulation experiments (MAEs). MAEs, combined with genomic sequencing, remain an accurate and widely used method to directly measure spontaneous mutation rates under minimal selection (Halligan and Keightley 2009; Lynch et al. 2016; Katju and Bergthorsson 2019). Selection is reduced during the experiment by imposing regular bottlenecks, generally at one cell or a mating pair, to decrease the population size of the MA lines. Such dramatic bottlenecks increase the drift to a level where selection is negligible. This allows the fixation of almost all but strongly deleterious mutations, and thereby gives access to the spontaneous mutation rate. However, for species with long generation times, a pedigree with parent–offspring genome sequencing is more appropriate and this approach has been employed frequently, notably in primates (Pfeifer 2017; Koch et al. 2019; Wang et al. 2020) and plants (Krasovec et al. 2018). To date, spontaneous mutation rates have been estimated in more than seventy species, including several groups of eukaryotes, bacteria, and archaea (supplementary table S1, Supplementary Material online). We observe a variation of four orders of magnitude between ciliates with *µ* = 7.6 × 10^−12^ (Long et al. 2018a) and the great apes, including humans, with *µ* = 1.3 × 10^−8^ mutations per site per generation (Besenbacher et al. 2016; Besenbacher et al. 2019). The “drift barrier” hypothesis has been widely accepted to explain such wide variation (Sung et al. 2012a; Lynch et al. 2016). According to this hypothesis, the mutation rate per site is lower in species with large effective population size (*N*_e_) because selection efficiently favors a small *µ*, which reduces the deleterious mutation load. However, in species with a small effective population size, *µ* cannot be maintained at a low rate because of stronger drift which can counteract selection. Current hypotheses on mutation rate variation are based on highly phylogenetically biased samples, particularly across eukaryotes. Indeed, the vast majority (>85%) of the estimates are based on only two eukaryotic groups: Opisthokonta and Archaeplastida (supplementary table S1, Supplementary Material online). Exceptions include one estimate for diatoms (Krasovec et al. 2019), four in alveolates (Sung et al. 2012b; Long et al. 2018a), one in haptophytes (Krasovec et al. 2020), and one in Amoebozoa (Saxer et al. 2012; Kucukyildirim et al. 2020). Interestingly, the mutation rates and spectra of these species are strikingly different when compared with classical plant and animal models. For example, the low mutation rates in *Paramecium* (Sung et al. 2012b; Long et al. 2015) may be explained by the unusual life cycle of ciliates, in which a transcriptionally silent germline genome undergoes rounds of cell divisions between sexual cycles. Selection would favor a low mutation rate to limit the number of deleterious mutations accumulated in the germline genome before sexual reproduction. In the case of *Dictyostelium discoideum*, the short indel mutation rate is higher than that for single nucleotide mutations (Kucukyildirim et al. 2020), contrary to observations in classical model organisms (Sung et al. 2016). In *Emiliania huxleyi*, the nucleotide mutation rate from GC to AT is lower than that from AT to GC (Krasovec et al. 2020), indicating that its mutational process tends to increase genome GC content, which is also the inverse of other studied eukaryotes. Altogether, these cases highlight that the diversity of mutation rates in eukaryotes is very likely underestimated. There is an enormous diversity of genome structures and life cycles across eukaryotes differing from classical biological models that may impact mutation rate evolution. Therefore, increasing the breadth of studied species across the tree of life is of critical importance to generate a more complete view of the causes, consequences, and evolution of mutation rates. In this context, the brown algae are a particularly interesting, albeit largely underexplored, group of eukaryotes. The brown algae, or brown seaweeds, are keystone species in today's ocean. They form underwater forests that provide the basis of exceptionally biodiverse ecosystem, and have a key role as carbon sinkers (Filbee-Dexter et al. 2022). Brown algae have been evolving independently from other eukaryotic multicellular groups (plants and animals) for more than a billion years (Coelho and Cock 2020), representing the third most developmentally complex multicellular lineage on the planet. Like most brown algae (Heesch et al. 2021), *Ectocarpus* has a haploid–diploid life cycle alternating between two independent, free living complex multicellular stages which are morphologically distinct: the gametophyte (haploid) and the sporophyte (diploid) (Bothwell et al. 2010; Coelho and Cock 2020). In the field, the two stages may inhabit different ecological niches and are often present during different seasons of the year (Couceiro et al. 2015) with a considerable portion of their life cycle spent in the haploid phase, potentially subject to haploid purifying selection (Immler and Otto 2018). Moreover, most brown algae, including *Ectocarpus* can reproduce both sexually and asexually (through spores produced from diploid sporophytes or by parthenogenesis via nonfertilized gametes which regenerate as haploid individuals), and some populations may reproduce almost exclusively asexuality (Couceiro et al. 2015). Consequently, these organisms supply an opportunity not only to study mutation rate evolution in a broad taxonomic context, but also to investigate the possible effect of a haploid–diploid life cycle that combines both successive asexual generations and the presence of a persistent haploid stage on mutation rate. Theoretical predictions suggest that such a life cycle may considerably reduce the mutation rate. First, asexual reproduction or reduced recombination are expected to increase the strength of selection for a low mutation rate because mutator alleles stay linked to a single lineage, increasing mutational load due to the accumulation of deleterious mutations over generations (Kimura 1967). This idea is supported by modeling approaches when drift has limited effect (Gervais and Roze 2017). Indeed the expected drift-barrier mutation rate is 1/*N*_a_ for asexual lineage with *N*_a_ being the asexual effective population size; and 1/2*N*_e_*s* for sexual species with *s* the mutation fitness effect (Lynch 2011). Second, a lower mutation rate would also be advantageous in this context, as an extensive haploid phase is expected to increase susceptibility to deleterious mutations and thus increase the haploid purifying selection efficiency against mutator alleles, contrary to animals with a gamete-limited haploid phase. In this study, we generated extensive genomic sequencing data from a pedigree of the model brown alga *Ectocarpus* (Coelho et al. 2012a; Coelho et al. 2020; Coelho and Cock 2020) and a hybrid cross of a sister lineage (*Scytosiphon*) to directly estimate the mutation rate and effective population size in representative members of the brown algae with a haploid–diploid life cycle. Our estimations suggest that the spontaneous mutation rate of these multicellular organisms is very low, on the order of mutation rates of bacteria or unicellular eukaryotes, whereas its effective population size is on the order of that of other multicellular organisms. We propose that the combination of haploid purifying selection and extensive asexual reproduction during the haploid–diploid life cycle of these organisms may contribute to their unusually low mutation rates.

## Methods

The *Ectocarpus* sp7 inbred lineage was generated by genetic crosses between siblings over eight meiotic generations from a wild type field collected diploid sporophyte (Ec17; supplementary fig. S1, Supplementary Material online). Note that *Ectocarpus* sp. (like most of brown algae) alternate between a diploid sporophyte which through meiosis produces male and female haploid multicellular gametophytes. The male and female gametophytes produce male and female gametes respectively, which after fusion reconstitute the sporophyte generation. There is no self-incompatibility in *Ectocarpus*, therefore sequential crosses between siblings to produce highly inbred lines is possible. We started a lineage from the field collected diploid sporophyte Ec17. We sequenced the genome of Ec372SP (Generation 0), and also four individuals from a first meiotic progeny of Ec372SP (Ec419f, Ec420m, Ec421f, and Ec423m, supplementary fig. S1, Supplementary Material online). Ec419f and Ec420m were crossed and used to continue the inbred line by crossing brothers and sisters at each generation. We then sequenced 30 haploid gametophytes produced by meiosis from the individual Ec467SP (supplementary fig. S1, Supplementary Material online). These 30 gametophytes were used to estimate the mutation rate. Note that for each inbred generation, gametophytes were isolated randomly out of several hundreds of gametophytes at a very early stage of development (a few cells stage), to ensure minimal selection.

DNA was extracted using the OmniPrep Plant kit (G-Biosciences) following the manufacturer's instructions. The DNAseq libraries were prepared following a polymerase chain reaction (PCR)-free protocol (Collibri PCR-free PS DNA library prep kit, ThermoFisher) and the genomic DNA of each individual was sequenced by Illumina NovaSeq with 150-bp paired-end reads. Raw reads were trimmed to remove poly(G) tails and overrepresented sequences with fastp (Chen et al. 2018) and TrimGalore (https://www.bioinformatics.babraham.ac.uk/projects/trim_galore/). Cleaned reads were then mapped onto the reference *Ectocarpus* genome (EctsiV2 from ORCAE database: https://bioinformatics.psb.ugent.be/orcae, (Sterck et al. 2012) with BWA-MEM (Li and Durbin 2010), and bam files were treated with samtools (Li et al. 2009). SNP calling to detect nucleotide and short insertion–deletion mutations was done with HaplotypeCaller from GATK (McKenna et al. 2010). *De novo* mutations candidates were identified based on several criteria. First, callable sites were considered if they had a coverage >9× in the parents Ec420m and Ec419f and the 30 progenies. Second, to decrease the rate of false positive mutations, only mutation candidates found in one single progeny individual were considered. We therefore discarded all variants shared within the 30 progenies, that may had arisen from standing genetic variation in the parents or *de novo* mutations appearing at any point between Ec372SP and Ec467SP. Mutations considered here are therefore only mutations appearing between Ec467SP and the 30 meiotic progenies, giving 30 progenies with one generation each, so a total of 30 generations in the whole experiment. This approach allowed us to use the 29 other progeny individuals to verify that the candidate is a true *de novo* mutation. Finally, the three individuals Ec372SP, Ec421f, and Ec423m were used as a means to further check *de novo* mutation candidates. Then, the following criteria were applied: 1) the alternative allele must be 100% of the coverage of the site to avoid somatic mutations: the genome is haploid so it is theoretically impossible to have a reference allele if the mutation was germinal; 2) none of the other individuals or parents must have any alternative reads even at low quality to avoid missmapping from repetitive sequences; and 3) mutation candidates were then checked: a) manually in the pileup file from samtools and by IGV (Robinson et al. 2011), and b) by PCR and Sanger sequencing. Note that we have tested if the mutation rate estimations would change (i.e., if we would recover more mutations) by changing the parameters and considering a mutation coverage of 90%. We do not recover more mutations by being less stringent, so we kept the 100% criteria.

Structural mutations in *Ectocarpus* were called with LUMPY (Layer et al. 2014), DELLY (Rausch et al. 2012), and SvABA (Wala et al. 2018), with the same identification criteria used for nucleotide and insertion–deletion mutations. In addition, candidates at a single position in a single individual were removed if similar variants were found near the position in other individuals. The mutation rate was calculated as follows: *n*/(G* × 30 × *g*) with *n* = the number of mutations, G* = the number of callable sites, 30 = the number of sequenced individuals after Ec467SP, and *g* = the generation number (*g* = 1).

To estimate the false negative rate, we simulated 100 *de novo* mutations in chromosome 20 of the reference genome and performed SNP calling. In brief, the simulation was performed by adding 100 mutations toward nucleotide G in the reference genome of the chromosome 20 from positions 60 to 76,860. The goal was to see if these artificial changes in the reference genome are detected and reported in the final vcf file. After modifying the reference, we mapped the reads of the individuals Ec420 with BWA and did the SNP calling with GATK HaplotypeCaller with the same parameters used in our mutation analysis. The mutated positions were then checked in the vcf file and the text format of the alignment (mpileup file from samtools). After ensuring that the mutations are correctly reported in the vcf and mpileup files, we started filtering the variants in our real data to detect only *de novo* mutations using the filters explained in the methods section. Ninety-seven of the 100 mutations were found, the three others were located in non-callable sites.

The coverage of whole genome of all individuals was calculated with bedtools (Quinlan and Hall 2010) by 5 kb windows to detect chromosome duplications. The chromosome Chr_00 of the assembly was ignored, because it is composed by all concatenated unmapped contigs.

To test the putative dosage compensation of duplicated chromosomes in the individual L467_27, we performed transcriptome analysis using triplicate samples of L467_27 with the line L467_26 as a control. Total RNA was sequenced by Illumina HiSeq with 100-bp paired-end reads. Raw reads were mapped against the reference transcriptome with RSEM (Li and Dewey 2011) with Bowtie2 (Langmead and Salzberg 2012) to get TPM values for each genes. Only genes with TPM higher than 1 were selected for analysis.

*Scytosiphon* cross is an interspecific cross between *Scytosiphon promiscuus* female (strain Mr5f) and *Scytosiphon shibazakiorum* male (strain Os10m) (supplementary table S2, Supplementary Material online). *S. promiscuus* and *S. shibazakiorum* are sister species with world-wide distributions (Hoshino et al. 2021), and have a haploid–diploid life cycle very similar to that of *Ectocarpus*, although the haploid phase is more conspicuous than the diploid phase (Heesch et al. 2021). Unilocular sporangia, where meiosis takes place, were isolated from the hybrid diploid sporophyte obtained from the hybrid cross. Similarly to *Ectocarpus*, each unilocular sporangium releases several hundreds of spores that grow into haploid multicellular gametophytes (Coelho and Cock 2020). One gametophyte per unilocular sporangium was randomly isolated and grown in standard brown algal culture conditions (Coelho et al. 2012b; Hoshino et al. 2019). In total, 137 F1 hybrid gametophytes and the two parents were sequenced to estimate the mutation rate. DNA was extracted using the OmniPrep Plant kit (G-Biosciences) following the manufacturer's instructions, and sequencing was performed by Illumina NextSeq 2000 with 150-bp paired-end reads. Raw reads were trimmed and filtered with Trimmomatic (Bolger et al. 2014). Because no reference genome was available, we performed a de novo assembly of the maternal strain Mr5f: filtered Illumina reads were assembled with Platanus Assembler (Kajitani et al. 2014). Bacteria contigs were identified using Blobtools (Laetsch and Blaxter 2017) and manually removed. Mutation identification was performed as described above for *Ectocarpus*, but with slight differences because of the lower average coverage of this genome. First, a coverage threshold of five or higher was used to consider a callable site. Second, because previously identified callable sites were not covered by five reads in all individuals, only sites covered by at least five reads in 137 to a minimum of 40 individuals and parents were selected (below 40 individuals the number of callable sites starts to decrease). Mutation rate was calculated as follow: *n*/(G* × *g*) with *n* = the number of mutations, G* = the number of callable sites, and *g* = the generation number (*g* = 1). Here, G* was calculated as follows: Σ(*i* × *N_i_*), where *i* (40 ≤ *i* ≤ 137) is the number of individuals and *N_i_* is the number of sites that have a minimum coverage of 5 in exactly *i* individuals. Because of the highly fragmented nature of the genome assembly, structural mutations were not called in *Scytosiphon*.

## Results

### Spontaneous Mutation Rate

**Fig. 1.**
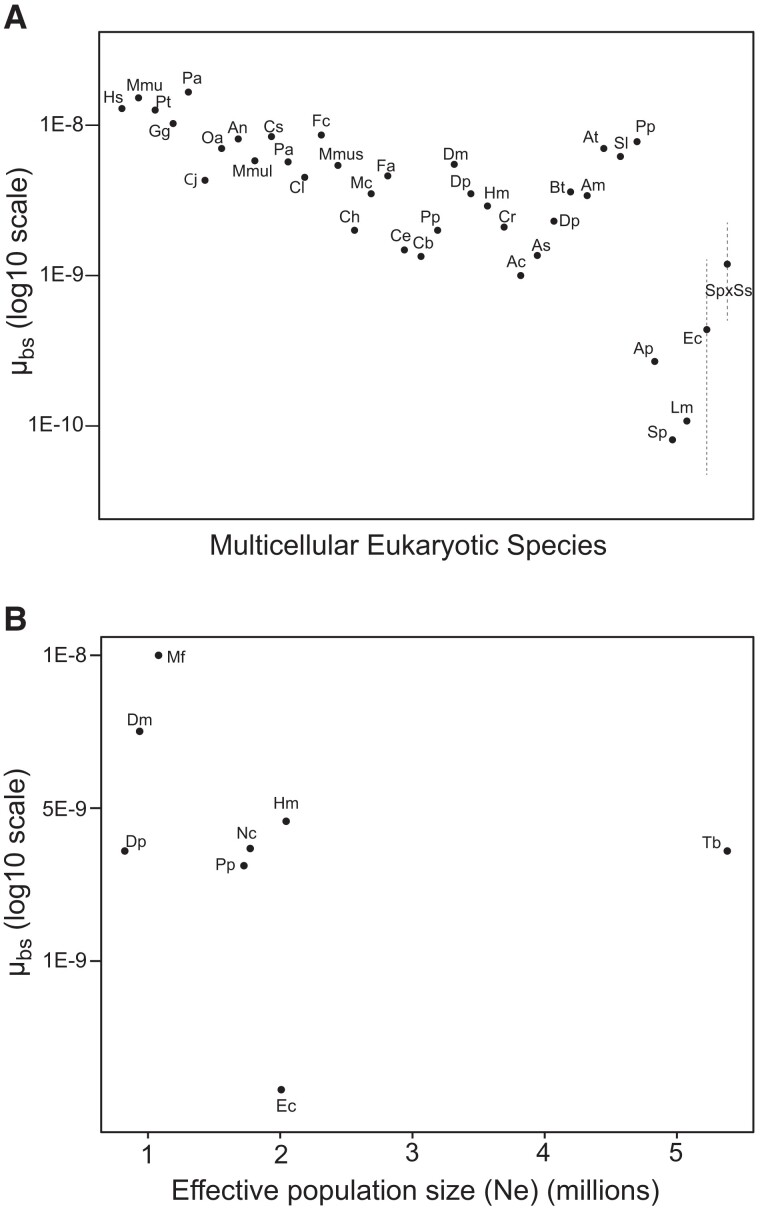
Nuclear nucleotide mutation rates *µ*_bs_ of multicellular species (see also supplementary table S1, Supplementary Material online). (*A*) Species left to right: *Homo sapiens*, *Microcebus murinus*, *Pan troglodytes*, *Gorilla gorilla*, *Pongo abeli*, *Callithrix jacchus*, *Ornithorhynchus anatinus*, *Aotus nancymaae*, *Macaca mulatta*, *Chlorocebus sabaeus*, *Papio anubis*, *Canis lupus*, *Felis catus*, *Mus musculus*, *Clupea harengus*, *Malawi cichlids*, *Ficedula albicollis*, *C. elegans*, *Caenorhabditis briggsae*, *P. pacificus*, *D. melanogaster*, *Drosophila pseudoobscura*, *H. melpomene*, *Chironomus riparius*, *Anopheles coluzzii*, *Anopheles stephensi*, *D. pulex*, *Bombus terrestris*, *Apis mellifera*, *Arabidopsis thaliana*, *Silene latifolia*, *Prunus persica*, *A. pisum*, *Spirodela polyrhiza*, *L. minor*, *Ectocarpus* sp.7, and *Scytosiphon* hybrid cross. Confidence intervals (CI) are represented for *Ectocarpus* (Ec) and *Scytosiphon* hybrid cross (SpxSs). (*B*) Nuclear nucleotide mutation rates *µ*_bs_ of species with effective population size of the same order as *Ectocarpus*, between 1 and 5 million (*P. pacificus*, *D. melanogaster*, *H. melpomene*, *D. pulex*, *N. crassa*, *M. florum*, and *T. brucei*). Effective population size from Lynch et al. 2016.

In *Ectocarpus*, we analyzed genomic data from the individual at the origin of the lineage (Ec372SP in supplementary fig. S1, Supplementary Material online), 2 parent individuals from the second generation (Ec419f and Ec420m), and 30 progenies. Mutations were called on 163,675,306 callable sites corresponding to 83.46% of the genome (supplementary table S2, Supplementary Material online). Two nucleotide mutations were identified (supplementary table S3, Supplementary Material online), 1 from A to G on chromosome 16 at position 1,499,719 (intergenic, individual L467_25) and 1 from C to A on chromosome 11 at position 645,251 (intron of the gene Ec-11_000650, individual L467_27). The mutation in chromosome 16 was validated by PCR and Sanger sequencing, but the mutation in chromosome 11 was located in a repeated region, precluding specific amplification and Sanger sequencing (supplementary table S4, Supplementary Material online). Both mutations were included in our estimates, to obtain a more conservative mutation estimation. The estimated nucleotide mutation rate was *µ*_bs_ = 4.07 × 10^−10^ (CI Poisson distribution: 4.93 × 10^−11^–1.47 × 10^−9^) mutations per site per generation, suggesting that *Ectocarpus* is one of the multicellular species with the lowest nuclear nucleotide mutation rate (supplementary table S1, Supplementary Material online, fig. 1). The relatively large confident intervals are unavoidable considering the low number of mutations obtained, but note even if the highest *µ*_bs_ value is taken, the mutation rate is still in the very low range for a multicellular eukaryote (fig. 1). We found no evidence for structural and short insertion–deletion mutations, indicating that the structural mutation rate *µ*_st_ of *Ectocarpus* may be even lower than *µ*_bs_.

In *Scytosiphon*, *de novo* genome assembly resulted in 159,922 contigs from 100 to 175,286 nucleotides for a total length of 185,347,032 nucleotides. The number of callable sites was variable within individuals (supplementary table S2, Supplementary Material online), from 162,402,871 to 523,289 with a total callable sites for the whole pedigree of 5,725,012,885 positions. Mutations were called on 107,429,108 sites corresponding to 57.96% of the genome. Seven de novo mutation candidates were identified and validated after PCR and manual IGV verification (supplementary table S3, Supplementary Material online, S4), giving a mutation rate for this hybrid cross of *µ*_bs_ = 1.22 × 10^−9^ (CI Poisson distribution: 4.92 × 10^−10^–2.52 × 10^−9^). The seven mutations include two transitions (C > T and T > C) and five transversions (A > T, G > T, C > A, T > G, and G > C).

### Chromosome Duplication in *Ectocarpus*

**Fig. 2.**
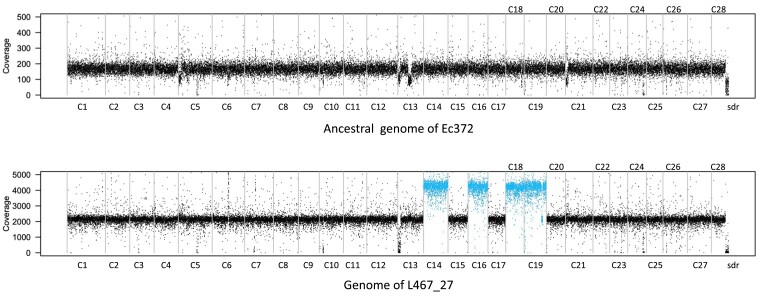
Whole genome raw coverage by 5 kb windows of the parental reference line Ec372 and L467_27 individuals, showing the chromosome duplications in L467_27 individuals. Grey bars mark chromosome separations. Ec372 is the ancestral genome. C1 to C28: chromosomes 1 to 28. sdr: sex determining region. Raw coverage of all individuals is provided in supplementary fig. S2, Supplementary Material online. The shared coverage variations within more than one line were not included in mutation rate calculation (e.g., the loss of coverage in the middle of chromosome 13 corresponding to the sex-specific region, supplementary fig. S2, Supplementary Material online). C: chromosome; sdr: female sex-specific contigs.

Assessment of raw genome coverage revealed a duplication of four chromosomes (C14, C16, C18, C19) in the individual L467_27 (fig. 2 and supplementary fig. S2, Supplementary Material online), that are covered by 2.0, 1.9, 1.9, and 2.1 of the non-duplicated chromosomes, respectively. The chromosome C19 of individual L467_31 has higher coverage than other chromosomes (supplementary fig. S2, Supplementary Material online), but by 1.8 only. In that last case, it is not possible to fully exclude an early somatic mutation shared by most of cells, where we do not count this event in the chromosome duplication rate. Assuming one independent whole genome duplication event, chromosome duplication rate is 0.0012 duplications per chromosome per generation or 0.033 chromosome duplications per cell per generation. Aneuploid karyotypes are usually highly deleterious (Sheltzer and Amon 2011), so we investigated if these chromosomal aberrations had an effect on individual fitness. Individual clone L467_27 was cultivated in standard culture conditions (Coelho et al. 2012b), and its development was closely followed by regular morphological measures (supplementary table S5, Supplementary Material online) and compared with a sibling without chromosome duplication (L467_26). After three weeks in culture, L467_27 exhibited an extensive decrease in fitness, with its growth being markedly slower (supplementary table S5, Supplementary Material online, supplementary fig. S3, Supplementary Material online). When cultivated in fertility-inducing conditions, control strain L467_26 produced reproductive structures (plurilocular sporangia) after 18 days, whereas L467_27 did not produce neither plurilocular sporangia nor meiotic reproductive structures (unilocular sporangia) even after 25 days. Taken together, these results suggest that the identified chromosome duplications did have a negative effect on the growth and reproductive fitness of this clone. To explore the effect of the whole chromosome duplications (WCDs) on transcription, we measured the level of genome-wide transcription of L467_27 and its sibling control (L467_26). Comparative transcriptomic analyses indicated a 1.71-fold higher mRNA level for genes located within the duplicated regions compared with non-duplicated regions (supplementary fig. S4, Supplementary Material online), suggesting a general lack of dosage compensation in the line harboring WCDs.

### Effective Population Size

Our estimate of the spontaneous mutation rate allows us to estimate the effective population size with *π*_s_ = 4 × *N*_e_ × *µ*. The neutral diversity (*π*_s_) of *Ectocarpus* has been estimated based on 49 sporophytes from three European and one South American populations and six gametophytes from two European and one South American populations (Avia et al. 2018). The neutral diversity of autosomal genes is *π*_s_ = 0.00323, which leads to an effective population size of ∼2 million. Note, however, that this *N*_e_ of 2 million is to be taken with caution because it is not known to what extent diversity may have been affected by demographic events in these populations. *Ectocarpus* has a haploid UV sex chromosome system (Ahmed et al. 2014; Coelho et al. 2018; Coelho et al. 2019), making it possible to calculate the effective population size of the different parts of the sex chromosome: *π*_s_ of the pseudo-autosomal region (PAR, the recombining part of the sex chromosome) is *π*_s_ = 0.0044 with *N_e_* ∼2.7 millions; and *π*_s_ of the sex-determining region (SDR, non-recombining) is *π*_s_ = 0.0022 with *N*_e_ ∼1.4 million. The higher *N*_e_ of the PAR region may be caused by balancing selection between male and female alleles (Avia et al. 2018), whereas the smaller *N*_e_ of the SDR is likely caused by the absence of recombination (Ahmed et al. 2014).

## Discussion

Our results reveal an unusually low spontaneous mutation rate in the model brown alga *Ectocarpus,* on the same order of magnitude as that of unicellular organisms such as bacteria (Long et al. 2018b), yeast (Lynch et al. 2008; Zhu et al. 2014), and phytoplankton (Ness et al. 2012; Krasovec et al. 2017; Krasovec et al. 2019; Krasovec et al. 2020). To date, estimates of mutation rates in multicellular species such as insects (Schrider et al. 2013; Keightley, et al. 2014a, 2014b; Liu et al. 2017; Oppold and Pfenninger 2017; Krasovec 2021), small vertebrates (Uchimura et al. 2015; Smeds et al. 2016; Feng et al. 2017; Malinsky et al. 2018), plants (Ossowski et al. 2010; Xie et al. 2016; Krasovec et al. 2018), and nematodes (Denver et al. 2009) are one order of magnitude higher. *Ectocarpus* is therefore one of the few cases of multicellular species with a such low spontaneous mutation rate (fig. 2*A*), although the upper limit of its mutation rate confidence interval is close to the smaller multicellular mutation rates. The mutation rate of the *Scytosiphon* hybrid cross reinforces the idea that these organisms may have very low mutation rates, considering this is a hybrid cross and hybridization and heterozygosity have been associated with exacerbated mutation rates is several species (Simmons et al. 1980; Yang et al. 2015; Xie et al. 2016; Krasovec 2021). Our observations therefore suggest that the selection pressure for a low mutation rate in brown algae may be stronger than for most of other multicellular species described so far. Following the drift barrier hypothesis effective population size is key to understanding mutation rate variation between species. Species with an effective population size on the same order of magnitude as *Ectocarpus* generally have a mutation rate one order of magnitude higher. For example, the effective population sizes of *Daphnia pulex*, *Drosophila melanogaster*, *Mesoplasma florum*, *Pristionchus pacificus*, *Neurospora crassa*, *Heliconius melpomene*, and *Trypanosoma brucei* are between 1 to 5 million, with spontaneous mutation rates from 1.4 × 10^−9^ to 9.8 × 10^−9^ mutations per site per generation. Species with a mutation rate similar to *Ectocarpus*, instead, have a larger effective population size of 10 million or more, notably unicellular species (Lynch et al. 2016). The low mutation rate of *Ectocarpus* is therefore unlikely to be explained by effective population size alone. There are two additional factors that, combined with effective population size, could potentially contribute to explain such low mutation rate. The first factor is asexual reproduction in this species via the production of mitotic spores or parthenogenesis in absence of gamete fusion (Bothwell et al. 2010; Couceiro et al. 2015; Coelho and Cock 2020). This hypothesis would be in line with the Kimura prediction (Kimura 1967), suggesting that the selection coefficient *k* for a low mutation rate increases when the recombination rate *r* decreases (*r* = 0 for asexual reproduction), given the general assumption of mutations as deleterious. Note that only two other examples of multicellular species with particularly low mutation rates have so far been described: the pea aphids *Acyrthosiphon pisum* (Fazalova and Nevado 2020) and the duck weeds *Lemna minor* (Xu et al. 2019; Sandler et al. 2020). These organisms can also reproduce asexually by budding and parthenogenesis for several generations before engaging in sexual reproduction, supporting the hypothesis that asexual reproduction indeed may drive the evolution of low mutation rates. The second factor is the haploid–diploid life cycle of *Ectocarpus* (and many other brown algae), which includes a persistent, complex, multicellular haploid stage. The gametophyte haploid stage of *Ectocarpus* has a relatively complex morphology, is free-living and macroscopic, persists for several months, and is associated with the expression of the majority of organismal genes (Lipinska et al. 2015). In contrast, the haploid stage of animals such as *Drosophila* is limited to the gametes, and in the diploid state, genetic dominance has the potential to mask the effects of mildly deleterious mutations. Thus, in *Ectocarpus* and other brown algae, haploid purifying selection may optimize selection against mutator alleles or any *de novo* mutations with effects on mutation rate (Kimura 1967).

WCD, which may result from a mis-segregation of the chromosome set between daughter cells, have been reported in several mutation accumulation studies in unicellular green algae (Krasovec et al. 2022) and yeast (Zhu et al. 2014; Liu and Zhang 2019). The chromosome duplication rate per cell we measured for *Ectocarpus* is several folds higher than in these species, although this rate should be taken with caution because of the limited data we have here. An explanation of high WCD rate compared with *µ*_bs_ is that mechanisms involved in WCD are very different than for other types of mutations. Although selection can act on several mechanisms to reduce nucleotide, short indel or structural mutation rates, chromosomes segregation are already highly optimized and under strong constraints and so possibly can no longer evolve towards a lower WCD rate. Aneuploid karyotypes are broadly considered deleterious because they cause an imbalance in gene dosage and transcript production (Hou et al. 2018). In *Caenorhabditis elegans*, excess transcripts from duplicated genes during MAE have been shown to be highly deleterious (Konrad et al. 2018). Mechanisms of dosage compensation, well known for sex chromosomes, may reduce the deleterious effects of such gene dosage imbalance by affecting the transcription rate (Disteche 2012). However, the relevance of dosage compensation mechanisms for autosomes, particularly immediately after duplication events is poorly understood. In this study, the reduced fitness of the individual with duplicated chromosomes could reflect a lack of dosage compensation, which was further supported by our transcriptomic analysis.

Together, our results suggest that organismal life cycle may play a significant role in mutation rate evolution and support models suggesting that asexuality and a persistent complex haploid phase may impact mutation rate. The mutation rate and effective population sizes estimations reported here will provide the basis for further studies on evolutionary processes in natural seaweed populations. More broadly, investigations of non-model or emergent model species will be key to improve our understanding of mutation rate evolution across the tree of life.

## Supplementary Material

msad105_Supplementary_Data

## Data Availability

Genomic raw read accession references are provided in supplementary table S6, Supplementary Material online.
