# Supplementary figures and images for: Low Spontaneous Mutation Rate in Complex Multicellular Eukaryotes with a Haploid–Diploid Life Cycle
Source: Mol Biol Evol. 2023 May 4;40(6):msad105. doi: 10.1093/molbev/msad105 (PMC10254074; doi:10.1093/molbev/msad105)

TPM ratio Ec467-27/Ec467-26

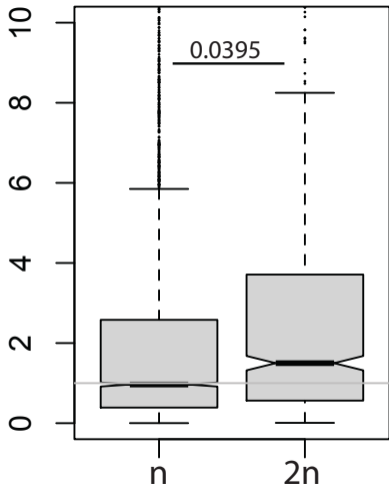

Supplement: msad105_Supplementary_Data [file msad105_supplementary_data.zip › Figure S4-R3.pdf]

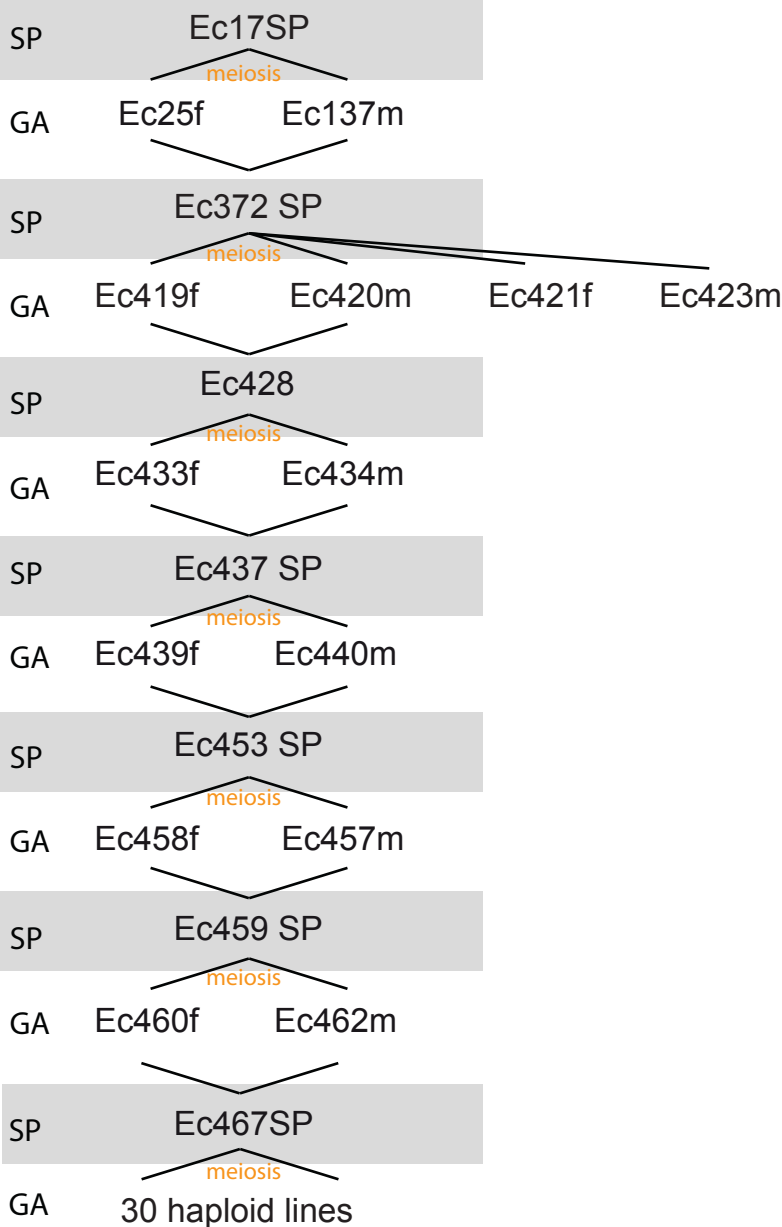

Supplement: msad105_Supplementary_Data [file msad105_supplementary_data.zip › Figure S1-R3.pdf]

L467\_27

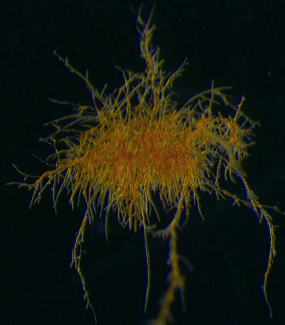

L467\_26

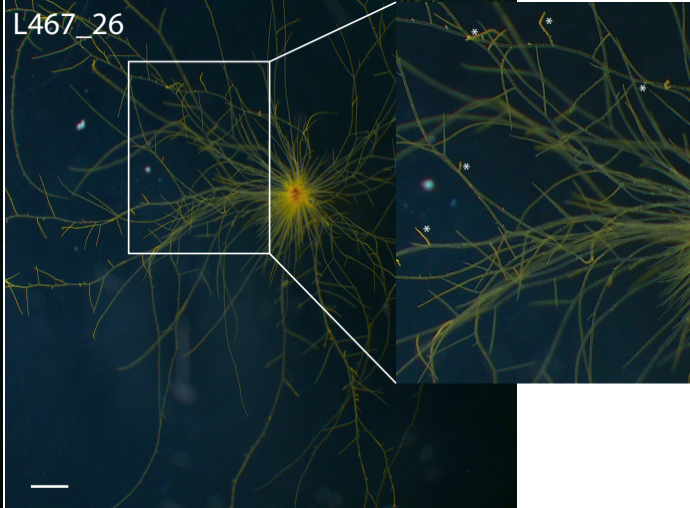

Supplement: msad105_Supplementary_Data [file msad105_supplementary_data.zip › Figure S3-R3.pdf]
